# *Ornithonyssus bacoti* (tropical rat mite): a critical review of biology, vector potential, and integrated management

**DOI:** 10.3389/fvets.2026.1954092

**Published:** 2026-10-13

**Authors:** Peng-Wu Yin, Xian-Guo Guo

**Affiliations:** Institute of Pathogens and Vectors, Key Laboratory of Ectoparasite Systematics and Evolution of Yunnan Provincial Education Department, Yunnan Provincial Key Laboratory for Zoonosis Control and Prevention, Dali University, Dali, China

**Keywords:** chemical control, morphological diagnostics, One Health, rodent mite dermatitis, vector competence

## Abstract

The tropical rat mite, *Ornithonyssus bacoti* (Hirst, 1913), is an obligate hematophagous gamasid mite of global veterinary, animal, and public health importance. While traditionally recognized as a cause of rodent mite dermatitis in humans, its potential involvement in zoonotic pathogen transmission requires rigorous critical evaluation. This paper provides a comprehensive review of research progress on *O. bacoti*, synthesizing evidence retrieved from major databases regarding its taxonomy, morphological diagnostics, life history, ecology, pathogen associations, genetic and physiological features, and integrated management. Morphologically, *O. bacoti* is distinguished from sibling species (*O. sylviarum* and *O. bursa*) by its long central dorsal setae, narrow tapering dorsal shield, and specific sternal plate setation. Under optimal laboratory conditions (28 °C, 75% relative humidity), its life cycle completes in 7–14 days, exhibiting arrhenotokous parthenogenesis. Epidemiologically, *O. bacoti* is a proven vector of *Rickettsia akari* (the causative agent of rickettsialpox) and serves as an intermediate host for the rodent filarial nematode *Litomosoides sigmodontis*. However, its role in transmitting Hantaviruses, *Bartonella* species, and other pathogens remains largely restricted to molecular detection or laboratory vector competence models, lacking definitive proof of natural transmission to humans. Furthermore, genomic insights remain preliminary, reliance on extrapolation from related poultry mites (*Dermanyssus gallinae*) must be carefully contextualized, and field resistance to pyrethroids warrants systematic monitoring. Effective mitigation requires a One Health strategy integrating host reduction, environmental sanitation, targeted acaricide use, and active vector surveillance.

## 1 Literature search strategy and inclusion criteria

To ensure reporting transparency and search reproducibility, a comprehensive and structured literature search was conducted across major electronic databases including PubMed, Web of Science, Scopus, Google Scholar, and the China National Knowledge Infrastructure (CNKI) covering records from 1913 through March 2026. The search strategy was restricted to English and Chinese peer-reviewed publications and employed standardized Boolean combinations of species identifiers (*Ornithonyssus bacoti*, *Liponyssus bacoti*, *Bdellonyssus bacoti*, and “tropical rat mite”) alongside topic-specific keywords (e.g., biology, morphology, life cycle, pathogen, dermatitis, genome, control, and acaricide).

Following initial identification, records underwent multi-stage screening. Duplicates were removed prior to title and abstract evaluation to filter out off-topic studies lacking diagnostic or comparative relevance to *O. bacoti*. Full-text articles were subsequently evaluated to exclude reports with ambiguous species identification or incomplete primary data. Ultimately, a refined cohort of 184 eligible studies was selected for final synthesis. To ensure analytical rigor, these publications were integrated using a three-tier level-of-evidence framework, categorizing insights into direct empirical evidence in *O. bacoti* (Level I), indirect evidence extrapolated from related mesostigmatid taxa (Level II), and biological hypotheses requiring further validation (Level III).

## 2 Discovery, nomenclature, and taxonomic status of *Ornithonyssus bacoti*

### 2.1 Discovery and nomenclature of *Ornithonyssus bacoti*

The tropical rat mite was first named in 1913 by the British zoologist Arthur Stanley Hirst. In 1912, G. Kristensen collected a large number of these gamasid mites from the body surface of rodents in Harar, Abyssinia (the former name of Ethiopia). Meanwhile, Dr. Petrie discovered and collected the tropical rat mite from the brown rat, *Rattus norvegicus* (formerly *Mus norvegicus*), captured in Assiut town, Egypt, but did not name it. Subsequently, Hirst conducted detailed observations and morphological descriptions of the mite based on female specimens obtained from Dr. Petrie, and named it *Leiognathus bacoti* Hirst, 1913 (1, 2). Later, based on the female, male, and nymphal specimens of the tropical rat mite collected from Australia and South America, Hirst redescribed the mite and noted that it might transmit the pathogens of plague or leprosy to humans (2).

### 2.2 Taxonomic status and synonyms of *Ornithonyssus bacoti*

With the advancement of mite taxonomy and the continuous discovery and nomenclature of new species, the taxonomic status of the tropical rat mite has undergone multiple shifts. At the family level, the tropical rat mite was successively assigned to different families, including Liponyssidae, Dermanyssidae, and Macronyssidae (3, 4). At the genus level, the tropical rat mite was previously placed into various genera, such as *Leiognathus*, *Haemogamasus*, *Liponyssus*, and *Bdellonyssus*. The major synonyms recorded in history include: *Leiognathus bacoti* Hirst, 1913; *Haemogamasus sanguineus* Ewing and Stover, 1915; *Liponyssus tenuiscutatus* Ewing, 1923; *Liponyssus nagayoi* Yamada, 1931; and *Bdellonyssus bacoti* (Hirst, 1913) Fonseca, 1941. Currently, the widely accepted scientific name for the tropical rat mite is *Ornithonyssus bacoti* (Hirst, 1913). The parentheses enclosing the author’s name and the year indicate that the species was not originally placed in the current genus *Ornithonyssus* when first described, but underwent subsequent generic revision (1, 3–6). According to the latest taxonomic system of gamasid mites, the taxonomic hierarchy of this mite is as follows: Phylum Arthropoda, Class Arachnida, Subclass Acari, Superorder Parasitiformes, Order Mesostigmata, Suborder Gamasina, Superfamily Dermanyssoidea, Family Macronyssidae, and Genus *Ornithonyssus* (7, 8, 86).

## 3 Morphological characteristics of *Ornithonyssus bacoti* and its differentiation from related species

### 3.1 Morphological structure and diagnostic features of *Ornithonyssus bacoti*

The body of the tropical rat mite, *Ornithonyssus bacoti*, is oval-shaped (Figure 1). Adult females reported in China measure 681–969 μm × 437–777 μm (9), although smaller individuals measuring 0.6–0.65 mm × 0.29–0.36 mm have also been recorded (10). In contrast, larger individuals have been documented in India, with an average length and width reaching 1.13 mm × 0.63 mm (11). These comprehensive literature records suggest substantial variations in the adult body size of *O. bacoti* across different regions worldwide. Males are generally slightly smaller and shorter than females, measuring 533–650 μm × 405–554 μm (Figures 2A,B).

**Figure 1:**
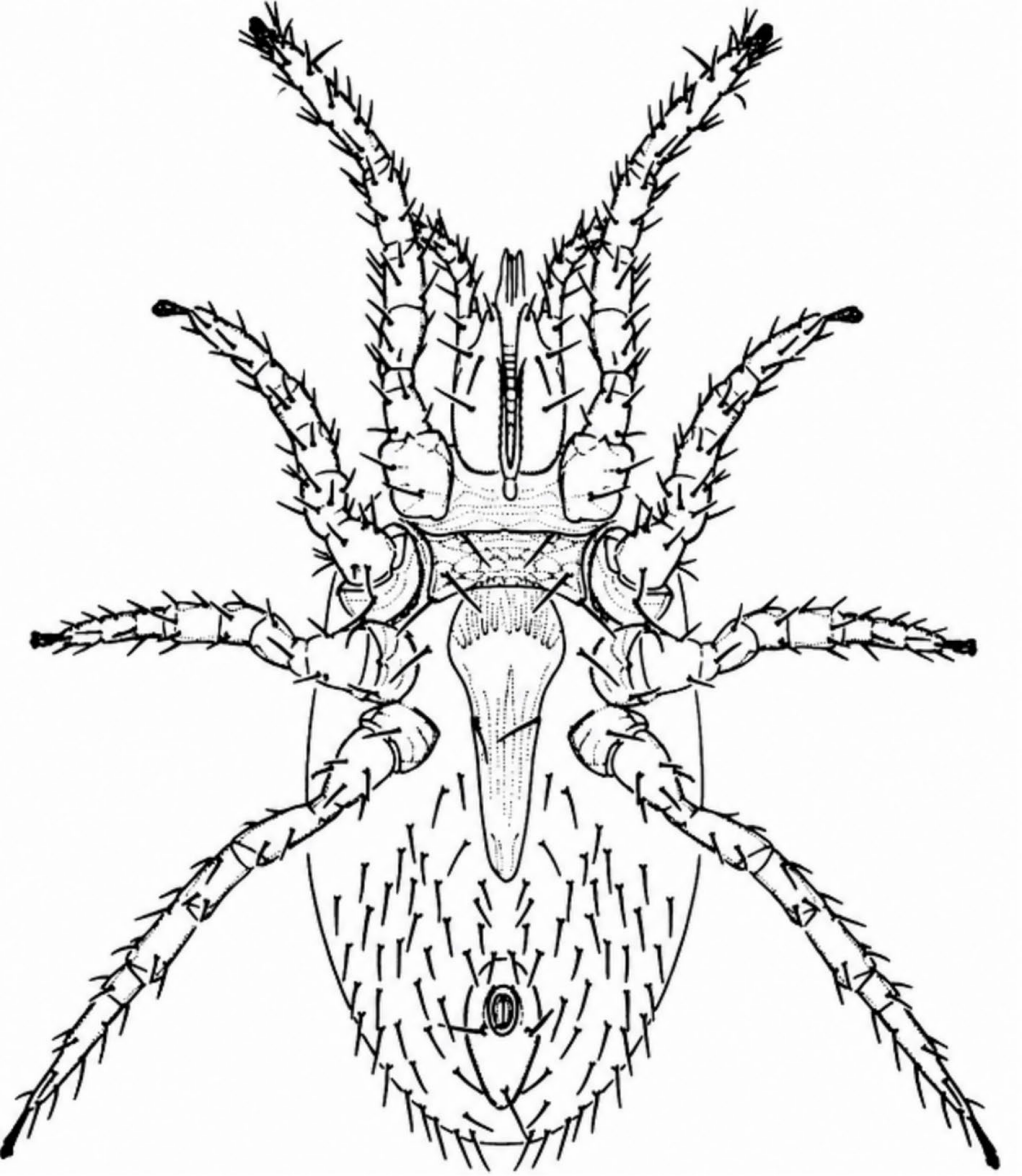
*Ornithonyssus bacoti*, female, ventral view [modified from Gorham (81)].

**Figure 2:**
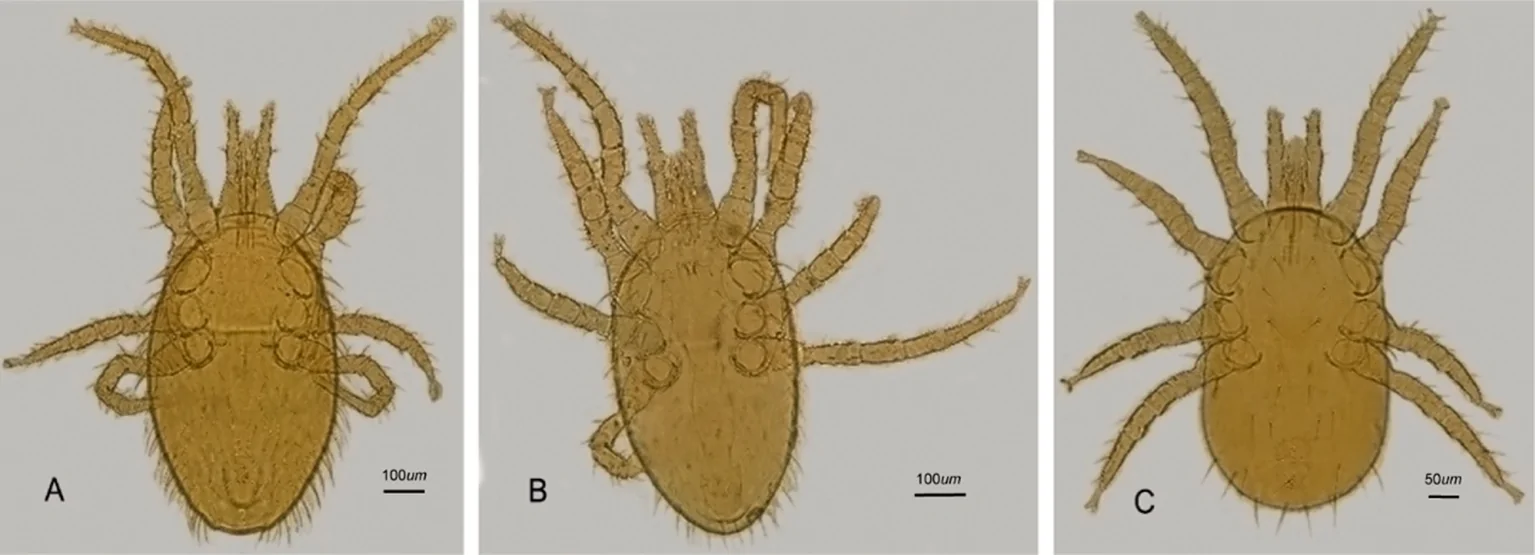
Photographs of tropical rat mites, *Ornithonyssus bacoti,* under a microscope (10 × 20; provided by Xian-Guo Guo). **(A)** Female adult; **(B)** male adult; **(C)** protonymph.

The idiosoma possesses an undivided, narrow, and elongated dorsal shield that covers only the anterior majority of the dorsal surface (Figure 2A). In adult females, the dorsal shield bears 27–28 pairs of setae in total. Conspicuously, the central dorsal setae are uniquely elongated, extending to or past the bases of the adjacent posterior setae, giving the mite a distinctly hairy appearance. The surrounding unsclerotized integument is densely covered with hypertrichous setae.

The gnathosoma clearly displays the chelicerae and palps (Figure 3). The chelicerae are relatively slender and shear-like, lacking internal denticles or cheliceral setae. The fixed digit is thinner than the movable digit. Males possess a spermatodactyl that is longer than the movable digit. Externally, the palps are slightly longer than the chelicerae. The idiosoma bears four pairs of legs, which are also covered with numerous setae. Within the deutosternal groove, there is a single longitudinal row of 10 denticles. The ventral anterior margin of the palpal trochanter bears a prominent process, and the palpal tarsal claw is bifurcated. The idiosoma narrows posteriorly starting from the level of coxa II (9–12).

**Figure 3:**
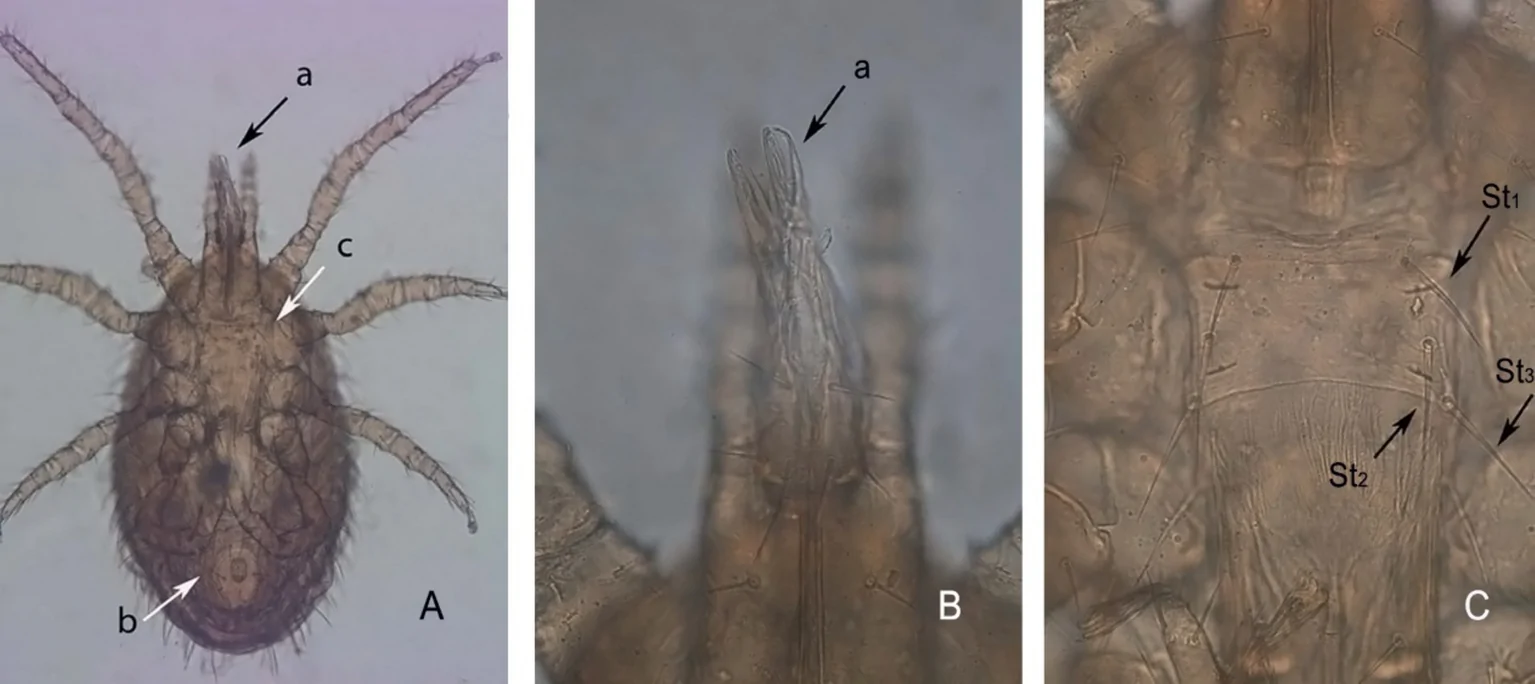
Morphological structure of *Ornithonyssus bacoti* [modified from Fiechter et al. (82)]. **(A)** Ventral view of female adult: a. chelicera; b. anal plate; c. sternal plate. **(B)** Chelicera of female adult: a. scissor-like end of chelicera. **(C)** Sternal plate with three pairs of setae (St_1_-St_3_).

### 3.2 Distinguishing morphological characteristics of *Ornithonyssus bacoti* and its closely related species

The primary sibling species of the tropical rat mite, *Ornithonyssus bacoti*, include *Ornithonyssus sylviarum* and *Ornithonyssus bursa*, among other congeneric species within the genus *Ornithonyssus*. *O. bacoti* primarily infests rodent hosts such as *Rattus norvegicus*, *Rattus tanezumi*, and *Mus musculus*. In contrast, *O. sylviarum* is predominantly associated with poultry and various wild birds, while *O. bursa* mainly parasitizes domestic poultry and wild birds, and other avian hosts. Although these three species exhibit high morphological similarity, they can be distinguished by the setae on the central region of the dorsal shield. In *O. sylviarum* and *O. bursa*, these central setae are relatively short, with their tips failing to reach the bases of the subsequent setae. Conversely, the central dorsal setae of *O. bacoti* are longer, extending to or beyond the bases of the following setae. Detailed morphological diagnostic keys and distinguishing characteristics of the three *Ornithonyssus* species are presented in Table 1 and Figure 4.

**Table 1:** Morphological differentiation of *Ornithonyssus bacoti* from other sibling mite species in the same genus.

Morphological features	*Ornithonyssus bacoti*	*Ornithonyssus bursa*	*Ornithonyssus sylviarum*
Central dorsal shield setae	Long; tips reach or extend beyond bases of adjacent posterior setae	Short; tips fail to reach bases of adjacent posterior setae	Short; tips fail to reach bases of adjacent posterior setae
Posterior end of dorsal shield	Gradually tapering to a narrow, pointed tip	Broader posteriorly, abruptly ending in a blunt tip	Moderately broad, ending in a blunt cone-shaped tip
Sternal shield setae count	3 pairs on the sclerotized shield	3 pairs on the sclerotized shield	2 pairs on shield; 3rd pair off shield on integument
Anterior margin of genito-ventral shield	With prominent median projection fitting sternal concavity	With broad median projection	With modest median projection
Primary host preference	Synanthropic and wild rodents (*Rattus* spp., *Mus* spp.)	Domestic poultry and wild passerine birds	Avian hosts (poultry, wild birds)

**Figure 4:**
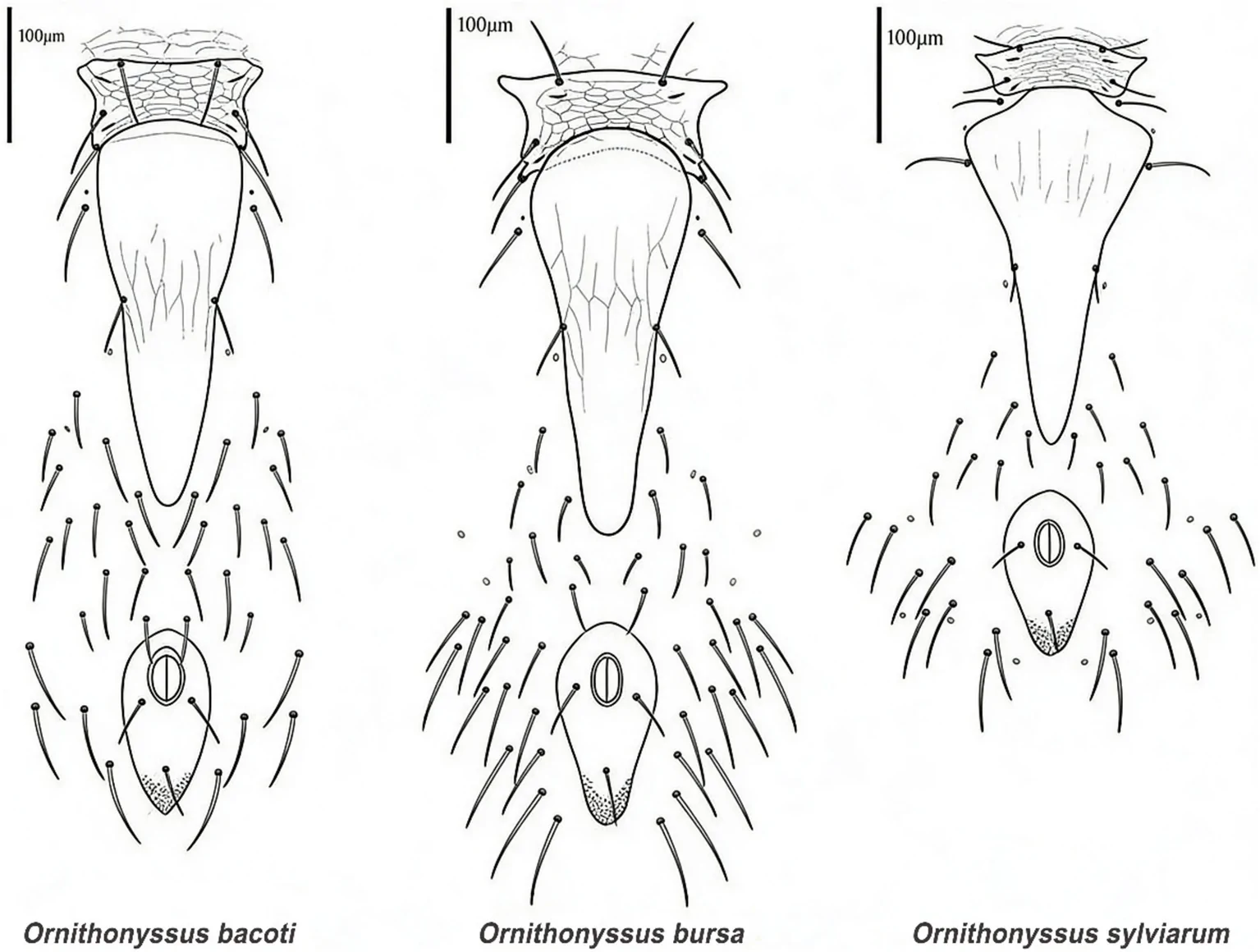
Morphological comparison on *Ornithonyssus bacoti* and other two sibling mite species in the same genus.

### 3.3 Ultrastructure of *Ornithonyssus bacoti*

#### 3.3.1 Dorsal shield

The dorsal shield covers the dorsum of the idiosoma and is scutiform (shield-shaped), with its surface densely covered by fine micro-pores and punctations. Spinose processes are visible along the lateral margins of the shield, which are hypothesized to enhance mechanical rigidity and assist the mite in anchoring itself between the host’s hairs. A characteristic arrangement of 11 setal pairs is conspicuously located on the posterior-lateral region of the dorsal shield, anchored by swollen bulbous bases that enhance mechanical flexibility during host movement. The bases of these setae are bulbously enlarged into swollen bases, forming a characteristic anchoring structure. This modification is believed to provide substantial gripping force when the mite traverses the dense pelage of its hosts (11, 13). Additionally, the micro-pores distributed across the dorsal shield surface are suggested to play a role in gas exchange or chemosensory functions (Figure 5).

**Figure 5:**
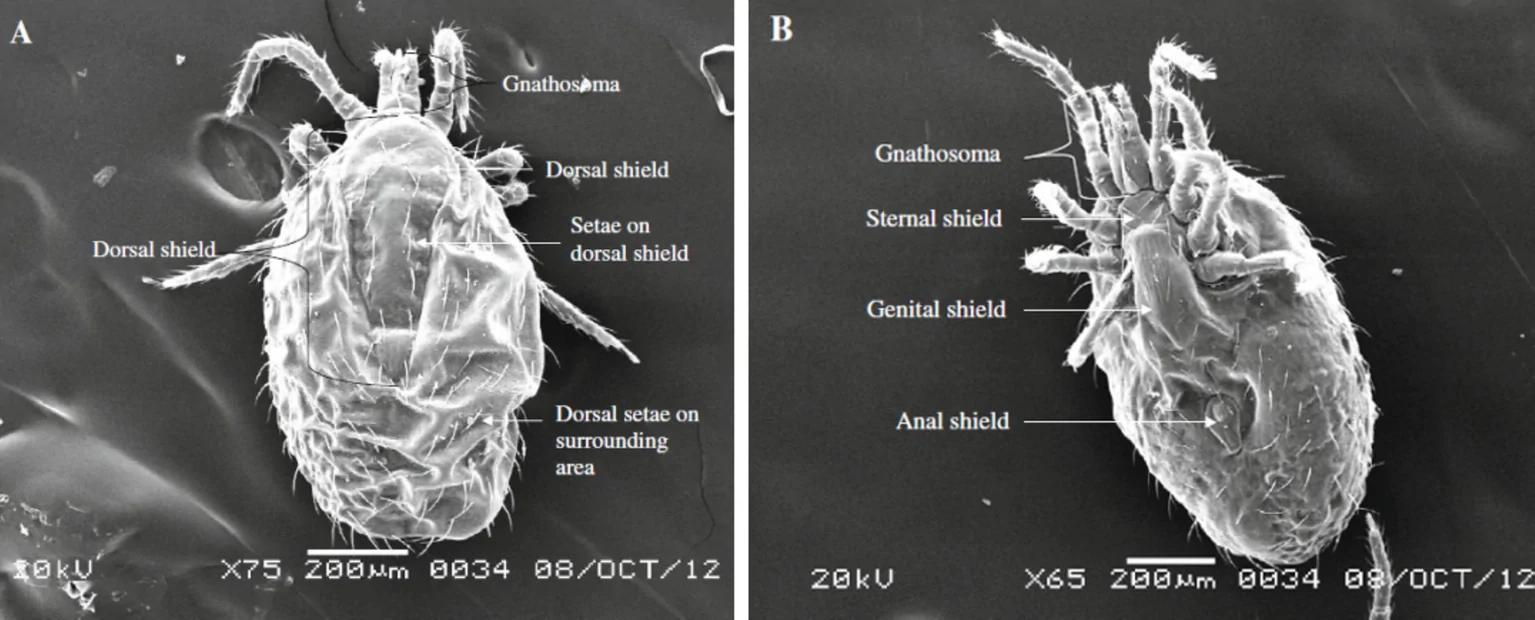
The dorsal view **(A)** and ventral view **(B)** of *Ornithonyssus bacoti* under the scanning electron microscope [modified from Nath et al. (11)].

#### 3.3.2 Peritreme and stigma

The stigma is positioned laterally between coxae III and IV, with a comma-shaped peritrematal plate extending anteriorly from the spiracle. The surface of the peritrematal plate is densely covered with minute micro-pores, which are speculated to participate in respiration or chemosensation. This structural feature was also documented by Fan et al. (14) during their survey of gamasid mites on rodents in Anhui Province of China.

#### 3.3.3 Legs

The distal ends of the legs are equipped with well-developed claws and pulvilli. Longitudinal striations are present on the claws to increase friction. The surface of the pulvillus is covered with microvilli, effectively expanding the contact area with the host’s hair shaft to enhance attachment. The flexible and elastic intersegmental membrane allows the pretarsus to adapt to host hairs of varying diameters and skin folds (15). All four pairs of legs (I–IV) are armed with prominent setae. Notably, the distal portion of tarsus I is heavily endowed with diverse sensilla, which are considered crucial for host-seeking behavior and chemoreception.

#### 3.3.4 Gnathosoma and mouthparts

Scanning electron microscopy (SEM) observations reveal that the gnathosoma and mouthparts of *O. bacoti* exhibit highly specialized morphological adaptations for an obligate hematophagous parasitic lifestyle. Located at the anterior apex of the body, the gnathosoma comprises the chelicerae, palps, and hypostome. The chelicerae are elongated and needle-like, containing internal spiral grooves that create a negative pressure channel during feeding, thereby facilitating rapid blood ingestion. The cheliceral digits bear minute apical serrations to penetrate the host’s epidermis (13). The distal end of the palp is differentiated into a palpal claw, surrounded by numerous sensory setae. Specifically, the palpal setae are golf-club-shaped, presumably functioning in tactile and chemical signal reception. Additionally, denticles are present on the ventral surface of the hypostome to assist in anchoring the mouthparts into the host’s skin.

#### 3.3.5 Sternal shield

The sternal shield is situated on the anterior region of the venter. It is morphologically flat and short, featuring a concave anterior margin and an almost straight posterior margin. The surface of the sternal shield is ornamented with fine punctations and bears three pairs of symmetric, trichoid sternal setae (S_t1_–S_t3_). Although the exact shape of the sternal shield exhibits minor inter- and intra-specific variations, its overall configuration remains stable, serving as an auxiliary character for species identification (11). Functionally, the sternal shield primarily serves as a site for muscle attachment and provides structural support for the body (Figure 6).

**Figure 6:**
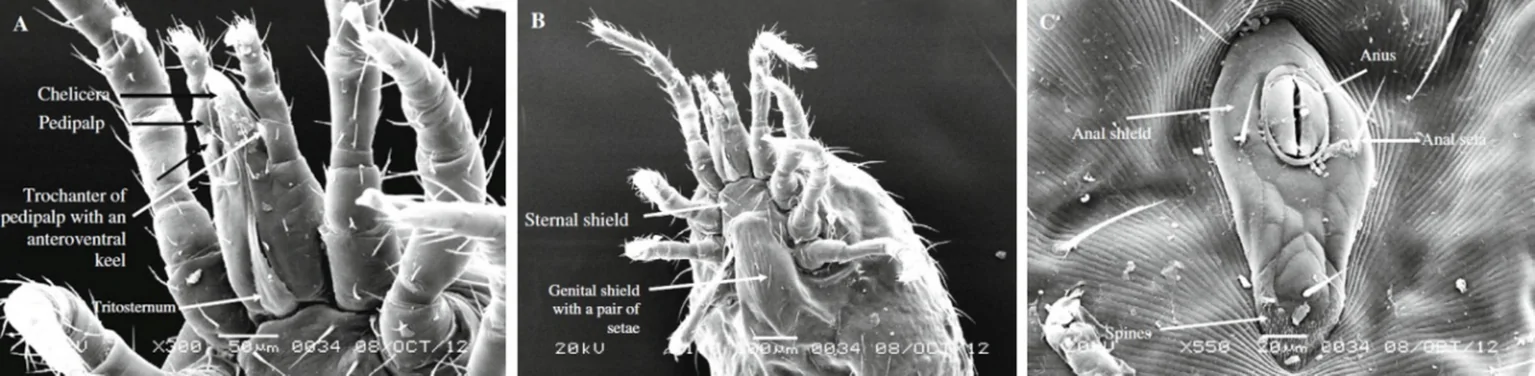
The gnathosoma **(A)**, sternal shield **(B)** and anal shield **(C)** of *Ornithonyssus bacoti* under the scanning electron microscope [modified from Nath et al. (11)].

#### 3.3.6 Anal shield

The anal shield is located on the posterior region of the ventral idiosoma and is pyriform (pear-shaped). The anal opening is situated cranially on the shield. A V-shaped cribrum consisting of spicules is arranged posteriorly, and three anal setae surround the anus. Sieve-like pores are visible on the shield surface, which are putatively associated with sensory or excretory functions. The configuration of the anal shield holds significant taxonomic value for interspecific differentiation within the genus (11, 16).

In summary, these ultrastructural features collectively demonstrate the exquisite morphological adaptation of *O. bacoti* to its obligate ectoparasitic lifestyle: the anchoring configurations of the dorsal shield and its setae ensure firm attachment within the host’s fur; the micro-pores on the peritrematal plate meet its respiratory demands; the finely differentiated chelicerae and palps facilitate highly efficient blood-feeding; and the structural stability of the sternal and anal shields provides reliable criteria for taxonomic identification.

### 3.4 Internal anatomy of the tropical rat mite (*Ornithonyssus bacoti*)

#### 3.4.1 Digestive system

The digestive system consists of three distinct parts: the foregut, midgut, and hindgut (Figure 7A). The foregut comprises the mouth, pharynx, and esophagus, with the muscular pharynx generating the suction required for blood-feeding. The midgut is the most prominent part and is divided into the anterior midgut and midgut caeca. The number and branching of the midgut caeca vary significantly depending on the nutritional status of the mite; they are small and flat when unfed but expand dramatically after blood-feeding, with the inner walls forming numerous folds to increase the absorptive surface area (16).

**Figure 7:**
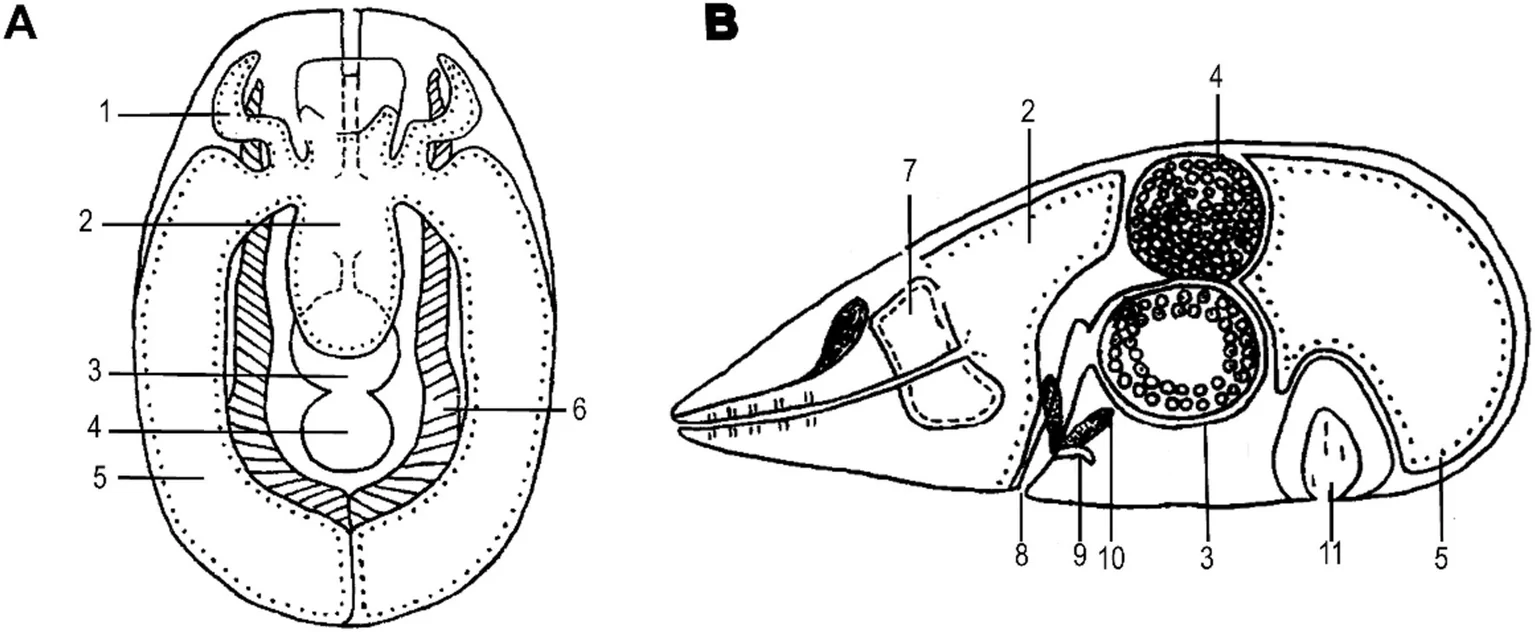
Schematic diagram of the internal structure of *Ornithonyssus bacoti* [modified from Zhuge and Meng (16)]. **(A)** Frontal view; **(B)** Sagittal plane. 1. Anterior caecum; 2. Midgut; 3. Uterus; 4. Ovary; 5. Posterior caecum; 6. Malpighian tubule; 7. Central nerve mass; 8. Gonopore; 9. Spermatheca; 10. Accessory gland; 11. Rectum.

Functionally, the midgut caeca are responsible for blood digestion and nutrient absorption. The increase in mucosal folds on the inner wall effectively improves the absorption efficiency per unit volume, enabling the mite to rapidly acquire sufficient nutrients from a single large blood meal to support subsequent development and reproduction. The hindgut is relatively short, connects to the rectum, and terminates at the anus. Secretory cells are present in the inner lining of the hindgut, participating in metabolic waste excretion and fluid reabsorption prior to elimination. These structural adaptations of the digestive system directly support the periodic blood-feeding behavior of *O. bacoti*.

#### 3.4.2 Reproductive system

The female reproductive system consists of a pair of tubular ovaries, oviducts, a uterine chamber (the terminal segment of the oviduct), a spermatheca, and a gonopore (Figure 7B). Oocytes at various developmental stages are visible within the ovaries, and the spermatheca is a sac-like structure. The male reproductive system comprises the testes, vas deferens, seminal vesicle, ejaculatory duct, and accessory glands surrounding the gonopore (16, 17).

Functionally, the female spermatheca stores sperm obtained from mating, enabling multiple fertilizations and batch oviposition. Oocytes progressively mature in the ovaries and are discharged through the oviducts. A single mating allows the female mite to oviposit multiple times, greatly enhancing its reproductive efficiency. The accessory glands surrounding the male gonopore likely secrete substances related to mating (such as spermatophore formation) to maintain sperm viability during transfer. This specialized reproductive structure enables *O. bacoti* to maintain a high reproduction rate in environments where host-switching is frequent.

#### 3.4.3 Nervous system

The nervous system exhibits a typical ladder-like arthropod structure, where the brain (synganglion) is formed by the fusion of the supraesophageal and subesophageal ganglia, surrounding the esophagus. The synganglion extends nerve branches anteriorly, which directly connect to the chemoreceptors at the base of the palps (16).

Functionally, the direct neural connection between the synganglion and the chemoreceptors enables the mite to rapidly respond to host chemical signals (such as CO₂, body temperature, and skin volatile organic compounds). This highly efficient signal transduction mechanism endows *O. bacoti* with precise host localization capabilities, significantly enhancing its parasitic success rate.

## 4 Life history of *Ornithonyssus bacoti*

The developmental life cycle of *Ornithonyssus bacoti* encompasses five distinct morphogenetic stages (Figure 8): egg, larva, protonymph, deutonymph, and adult (18). Under optimized laboratory regime (28 °C and 75% relative humidity), complete egg-to-adult development is achieved within 7–14 days. Thermal reaction norms indicate that developmental rates decline rapidly below 20 °C, whereas ambient temperatures exceeding 35 °C or relative humidity falling below 50% markedly elevate stage-specific mortality (12, 15).

**Figure 8:**
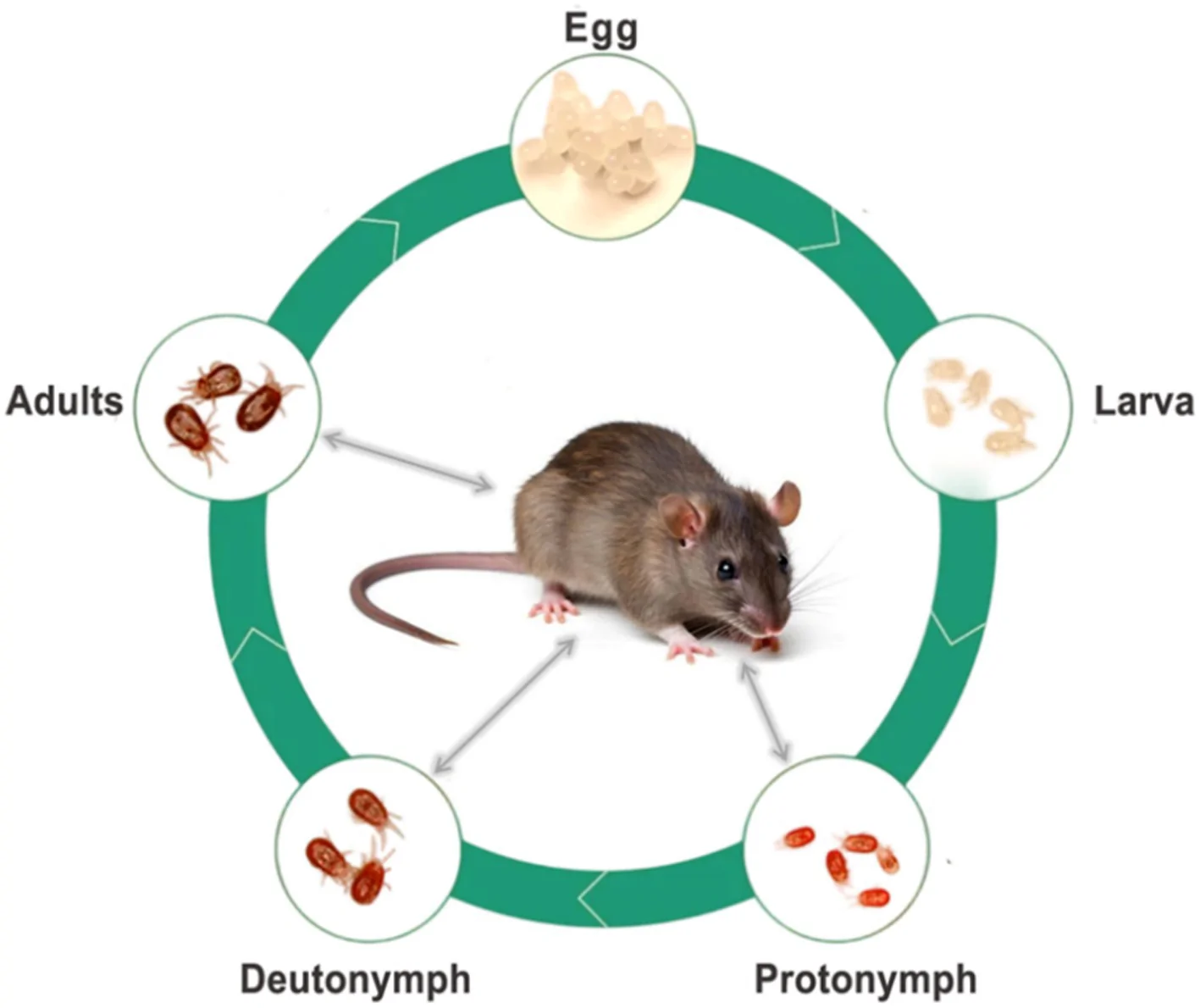
The life cycle of *Ornithonyssus bacoti* (provided by Peng-Wu Yin**).** Annotation: Development from egg to adult requires only 7–14 days, with optimal conditions at 28 °C and 75% relative humidity.

Fecundity is characterized by an average lifetime output of 50–100 eggs per engorged female, with peak daily oviposition reaching up to 20 eggs (19). Oviposition predominantly occurs off-host within host nest substrates and structural crevices. Reproduction follows an arrhenotokous parthenogenetic system, wherein unfertilized haploid eggs yield male progeny (*n* = 8) and fertilized diploid eggs produce females (2*n* = 16) (20). Obligate hematophagy is required for both nymphal instars (protonymph and deutonymph) and adults to progress through molting and reproduction, respectively, whereas the hexapod larval stage remains non-feeding (15, 19).

## 5 Ecological studies of *Ornithonyssus bacoti*

### 5.1 Behavioral dynamics and host-seeking mechanisms

The behavioral habits of the tropical rat mite (*O. bacoti*) demonstrate a high level of adaptation to host environments and a diversity of survival strategies. In terms of feeding and blood-sucking behavior, *O. bacoti* is an obligate hematophagous ectoparasite (Figure 9). The behavioral repertoire of *O. bacoti* reflects specialized evolutionary adaptations to a nidicolous, hematophagous lifestyle. Host-seeking exhibits a marked nocturnal periodicity driven by negative phototaxis and positive thermotaxis. Mites utilize specialized tarsal sensilla on leg I to perceive chemical cues, notably host skin volatiles, carbon dioxide gradients, and body heat, enabling precise host location over short distances (15). In the absence of primary rodent hosts, starving protonymphs and adults display active dispersal behaviors, migrating along architectural conduits, piping, and floor crevices (12). Off-host survival during starvation spans several weeks under humid conditions, facilitating host-switching. Furthermore, females display strong thigmotactic aggregation, clustering tightly within dark nest microhabitats to deposit eggs, thereby minimizing desiccation risk (15, 20, 21).

**Figure 9:**
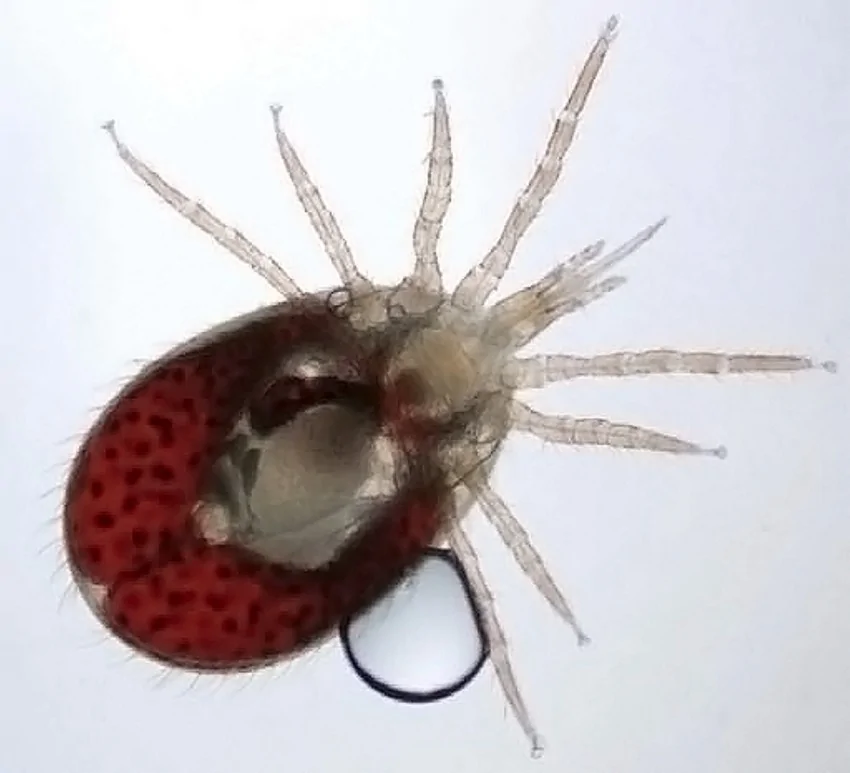
A blood-engorged female *Ornithonyssus bacoti* [modified from Hill et al. (77)].

### 5.2 Geographical distribution and host selection of *Ornithonyssus bacoti*

The global distribution of *O. bacoti* is characterized by both widespread prevalence and regional specificity. To rigorously re-evaluate the global range of *O. bacoti*, we synthesized verified georeferenced records from international repositories (e.g., GBIF) and localized epidemiological surveys (Figure 10). Contrary to informal assertions of a uniform cosmopolitan distribution, confirmed occurrence records reveal a highly structured pattern closely linked to synanthropic rodent host dissemination, urbanization, and international commerce.

**Figure 10:**
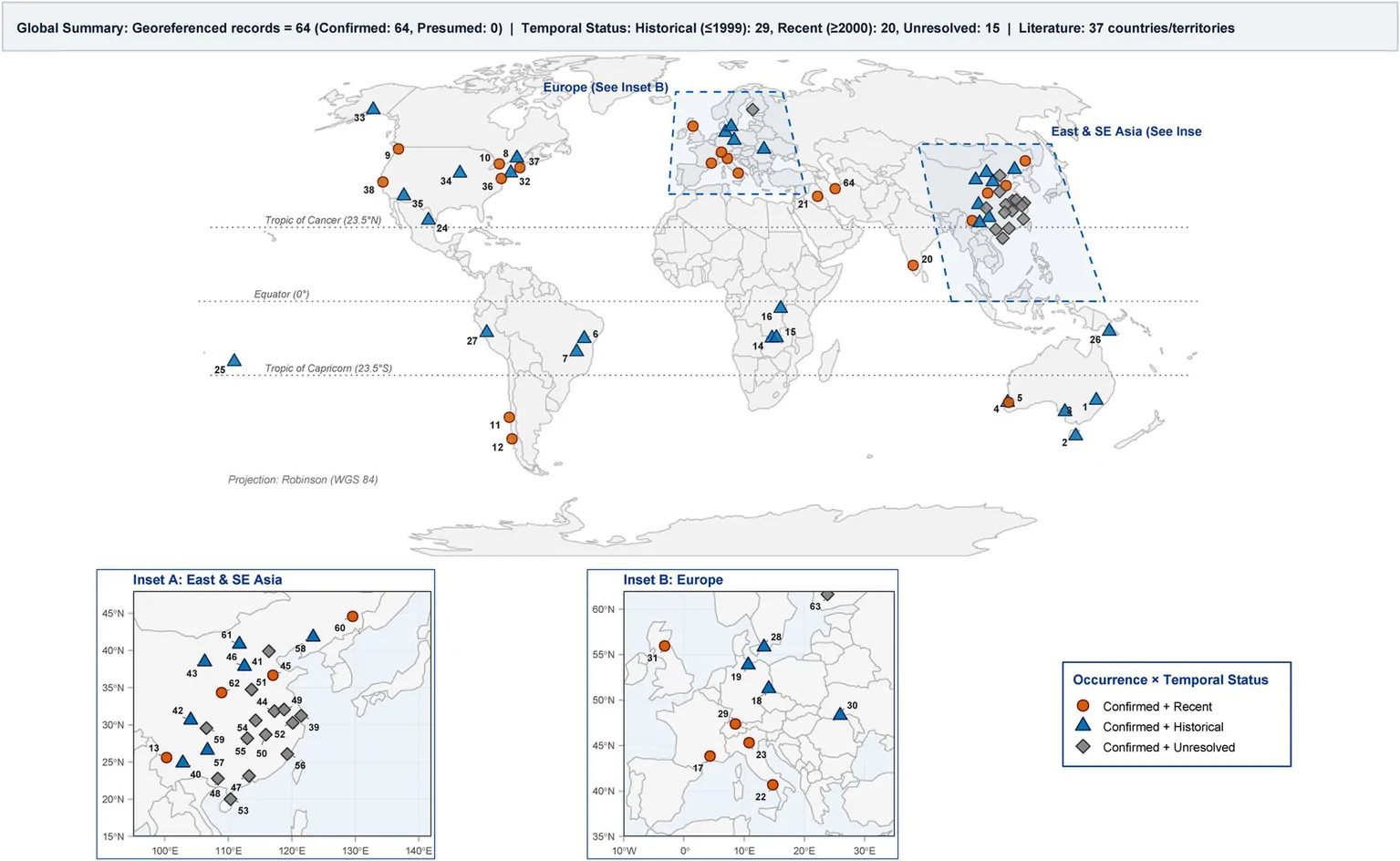
Global georeferenced occurrence and biogeographic distribution pattern of *Ornithonyssus bacoti*. Annotation: Spatial distribution of 64 confirmed georeferenced records compiled from published literature and open-access databases. Base map is presented in a Robinson projection. Dashed horizontal lines represent the Equator (0°) and the Tropics of Cancer and Capricorn (23.5°N/S), highlighting the predominantly tropical/subtropical core range and temperate synanthropic extensions. Red circles denote confirmed occurrences recorded from 2000 onwards (Recent); blue triangles indicate historical records prior to 2000 (Historical); gray diamonds mark confirmed records with unresolved sampling dates. Point IDs correspond to Supplementary Table 1 (prefixed with “OB-”). To resolve high-density spatial clustering, regional details are provided in Inset A (East & Southeast Asia) and Inset B (Europe). Records at extreme northern latitudes (e.g., location ID 33 in Canada and 63 in Northern Europe) represent indoor/port synanthropic occurrences constrained by low-temperature developmental limits of the mite and its primary rodent hosts (*Rattus* spp.). Country-level presumed occurrences lacking precise coordinates (French Polynesia, Russia, Ukraine, Slovakia) are detailed in Supplementary Table 1.

Confirmed occurrences span tropical, subtropical, and temperate zones across six continents, with major clusters documented in North America, East Asia, Western Europe, and coastal Australia (Figure 10). Stratification by timeline demonstrates that while historical records (≤1999) established its presence along maritime trade routes and primary port cities, recent records (≥2000) show expanding detection in indoor urban environments, laboratory animal facilities, and suburban residential zones. Presumed occurrences remain concentrated in rural or under-surveyed regions where primary hosts (such as *Rattus norvegicus* and *Rattus tanezumi*) are abundant but specialized acarological monitoring is lacking. *O. bacoti* remains undetected in polar regions and extreme high-altitude alpine zones, constrained by environmental temperatures unsuitable for off-host survival and development.

Although its native distribution is closely tied to the dispersal of rodent hosts (e.g., *Rattus norvegicus*), it has spread to most temperate and tropical regions worldwide through host migration and human activities, such as port trade and laboratory animal transportation (15). From the perspective of zoogeographical regions, this mite is widely distributed in the Palearctic (Europe, North Africa, and Northern Asia), Nearctic (North America), Oriental (Southeast Asia), and Neotropical (Central and South America) regions, whereas records in the Afrotropical and Australasian regions are sparse, which may be attributed to host limitations or insufficient surveillance (12, 18). Notably, its distribution is closely linked to climatic conditions, as the species is highly adapted to warm and humid environments (annual mean temperature of 15–30 °C and annual precipitation of 500–1,500 mm). For instance, its population density in India, Southern China, and Southeast Asia is significantly higher than that in arid or cold regions (11, 22).

Furthermore, anthropogenic activities have accelerated the trans-regional transmission of *O. bacoti*, leading to localized outbreaks among rodent populations in non-native areas such as Northern Europe and Canada (20). Its prevalence in urban environments is particularly pronounced, which is directly associated with the high spatial overlap between synanthropic rodents (e.g., *R. norvegicus*) and human residential areas (23). However, despite its extensive distribution, records from polar and high-altitude regions (e.g., the Qinghai-Tibet Plateau and the Arctic Circle) are extremely scarce, indicating that low temperatures or extreme climates impose critical limits on its survival (12).

Meanwhile, host preferences of *O. bacoti* vary among different geographical populations. For example, North American populations of the mite more commonly parasitize sciurid hosts (squirrels), whereas Asian populations utilize *R. norvegicus* as their primary host (18). These characteristics indicate that the dispersal capacity of *O. bacoti* depends on both host ecological niches and the intensity of human activities, while also being naturally constrained by climate and geographical barriers. Overall, while its global transmission risk is exacerbated by urbanization and international trade, its distribution in high-latitude and high-altitude areas remains limited by environmental tolerance.

As an obligate hematophagous ectoparasite, *O. bacoti* possesses a broad host range, primarily parasitizing various rodent species, among which *Rattus norvegicus* and *Rattus tanezumi* serve as the main hosts. Additionally, this mite can parasitize a variety of wild small mammals, including *Bandicota indica*, *Apodemus agrarius*, *Bandicota bengalensis*, and even birds. In the absence of suitable hosts, it may actively bite humans (15, 18, 23).

### 5.3 Population ecology of *Ornithonyssus bacoti*

The population ecological characteristics of *O. bacoti* are closely tied to its hosts and environmental conditions. Its spatial distribution is highly aggregated, primarily concentrated in host nests and high-activity zones (such as laboratory animal facilities or urban sewers), and the degree of mite aggregation increases significantly with higher host densities (11, 18). Parthenogenesis is a prevalent gender determination mechanism in *O. bacoti*. Field investigations in southwestern China indicated a female-biased sex ratio (3:1 to 5:1) among gamasid mites collected from wild rodents, a dynamic governed by arrhenotokous parthenogenesis under selective pressure rather than an invariant physiological constant (24–27).

Regarding age structure, nymphs account for the highest proportion (approximately 50–60%), followed by adults (30–40%), and larvae are the least abundant, indicating that the nymphal stage is the critical period for population expansion (22).

The population dynamics of *O. bacoti* are jointly driven by host abundance, environmental conditions (e.g., temperature and humidity), and human activities. Its broad host adaptability and aggregated distribution strategy enable it to dominate specific ecological niches, which concurrently increases the difficulty of controlling its transmission. Therefore, effective prevention and control must integrate comprehensive measures, including host management (rodent control), environmental intervention (dehumidification), and scheduled chemical treatments (11, 15).

### 5.4 Other ecological studies of *Ornithonyssus bacoti*

The interspecific relationships between *O. bacoti* and other ectoparasites are primarily characterized by niche differentiation and competitive coexistence. Research indicates that there are spatial distribution differences on the host’s body surface between *O. bacoti* and other common rodent ectoparasites, such as *Xenopsylla cheopis* or *Polyplax spinulosa*. While *O. bacoti* prefers the host’s back and thin-skinned areas of the abdomen, *X. cheopis* tends to cluster around the ears and tail. This spatial segregation likely minimizes direct competition (28). Furthermore, in laboratory or urban settings, *O. bacoti* and other species of the genus *Ornithonyssus* (e.g., *Ornithonyssus sylviarum*) exhibit host selection differentiation; the former prefers rodents, whereas the latter primarily parasitizes birds, thereby reducing interspecific resource overlap (29). This partitioning mechanism further supports the applicability of niche theory to ectoparasite communities.

Studies have found that *O. bacoti* may affect the population dynamics of other parasites through indirect competition. Its high reproductive rate and wide host adaptability can compress the survival space of sympatric gamasid mites, especially when host densities are low (30). Concurrently, *O. bacoti* serves as a confirmed biological vector for *Rickettsia akari* and may act as an opportunistic mechanical carrier or laboratory host for other agents (such as Hantaviruses), potentially participating in ectoparasite-pathogen interactions alongside sympatric ticks or fleas (23). However, its public health importance for viral transmission remains secondary to aerosol pathways, requiring strict differentiation between proven vector competence and incidental pathogen carriage.

Remarkably, there is no direct competition between *O. bacoti* and other synanthropic mites in human environments, such as *Dermatophagoides* spp., due to their significantly different ecological requirements: the former relies on host animal blood, while the latter feeds on dander or organic debris (20). However, in specific scenarios (such as warehouses or poultry farms), *O. bacoti* may exhibit alternating outbreak patterns with other hematophagous mites, such as *Dermanyssus gallinae*, with their population fluctuations jointly regulated by host migration and insecticide usage (31, 32). This demonstrates that the interaction between environmental factors and parasitic adaptability plays an important role in shaping interspecific relationships.

In conclusion, the interactions between *O. bacoti* and other ectoparasites are dominated by niche differentiation and indirect competition, where differences in host preferences, spatial distributions, and drug resistance serve as key mechanisms maintaining coexistence. Nevertheless, its potential synergistic role in pathogen transmission warrants further research, especially regarding its dynamic fluctuations in complex ecosystems and corresponding public health risk assessments (23, 32).

## 6 Relationship between *Ornithonyssus bacoti* and diseases

### 6.1 Clinical manifestations of rat mite dermatitis and differential diagnosis

When primary rodent hosts are depleted or excluded, hungry protonymphs and adult *Ornithonyssus bacoti* actively migrate to seek human hosts. Biting produces rodent mite dermatitis (gamasid dermatitis), clinically characterized by intensely pruritic, erythematous papules, papulovesicles, or urticarial plaques measuring 3–10 mm in diameter. Lesions are predominantly concentrated on skin areas exposed during sleep or constrained by tight clothing, such as the waist, abdomen, shoulders, thighs, and flexoral folds, while scalp involvement is exceptionally rare. Differential diagnosis requires careful exclusion of other arthropod bites and dermatoses: unlike bedbug (*Cimex lectularius*) bites which often present in linear clusters (“breakfast, lunch, and dinner” pattern), *O. bacoti* lesions are randomly scattered; unlike scabies (*Sarcoptes scabiei*), *O. bacoti* does not construct stratum corneum burrows; and unlike flea bites, which concentrate heavily on the lower legs, *O. bacoti* lesions target covered trunk areas. Evidence-based clinical management relies on topical mid-to-high potency corticosteroids and oral antihistamines to diminish hypersensitivity reactions. However, pharmacological intervention is strictly symptomatic; complete resolution necessitates concurrent rodent reservoir eradication and residual environmental acaricidal decontamination.

### 6.2 Direct harms of *Ornithonyssus bacoti*

*Ornithonyssus bacoti* can cause gamasid dermatitis (also known as rodent mite dermatitis) through biting humans, with its direct harms primarily manifesting as skin lesions and systemic symptoms (Figure 11). Following mite bites, erythematous papules (typically 3–10 mm in diameter) frequently develop on the patient’s skin, which are sometimes accompanied by a surrounding halo, and occasionally vesicles or nodules. These lesions are mostly distributed on exposed body parts such as the limbs, shoulders, back, and waist, while the scalp is rarely involved (11, 33). In addition, intense pruritus is the core symptom, which typically worsens at night and may lead to secondary infections or epidermal excoriation due to scratching (20, 34).

**Figure 11:**
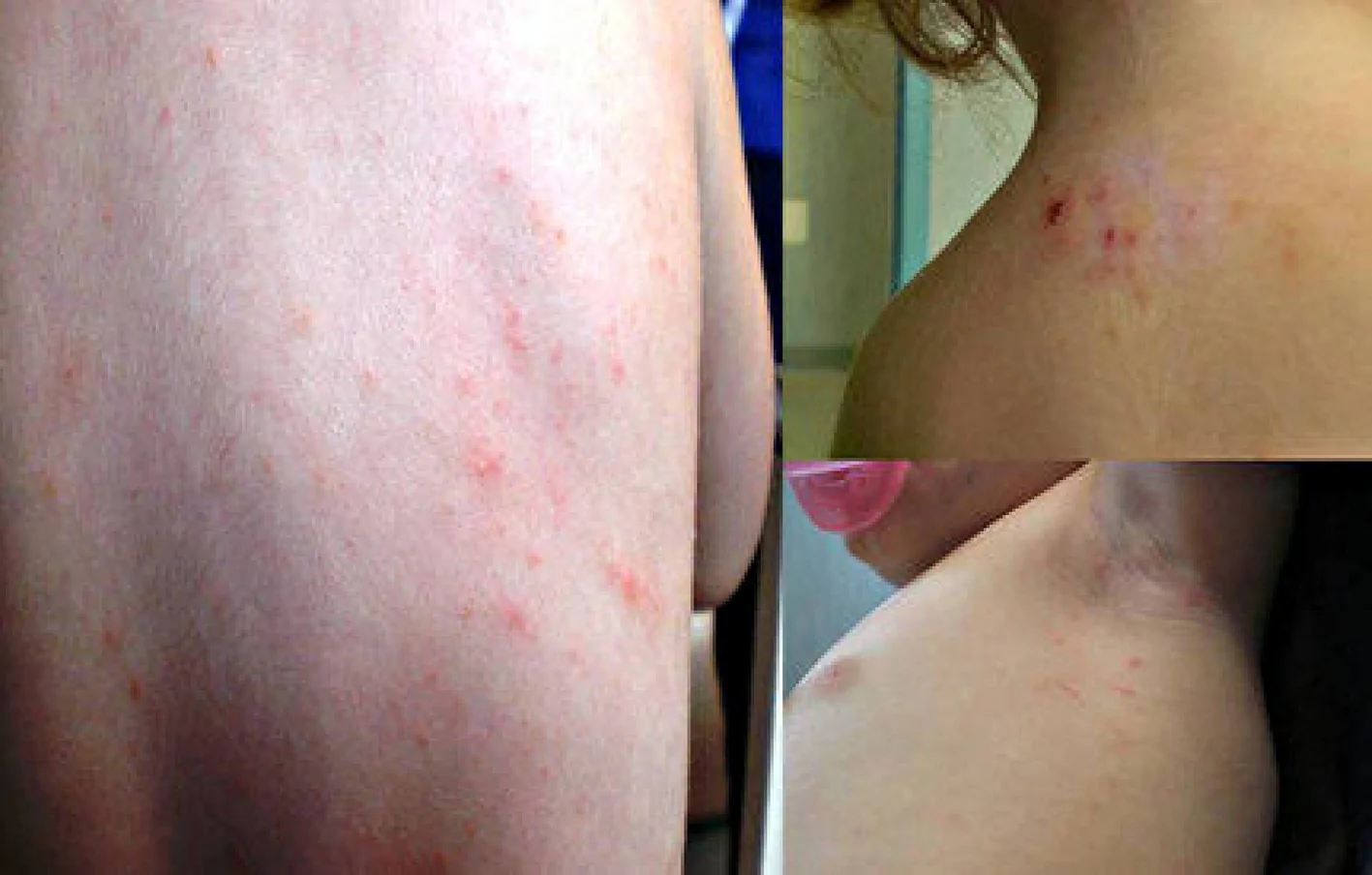
Skin lesions of humans caused by the biting of *Ornithonyssus bacoti* [modified from Nath et al. (11)].

Studies have shown that this dermatitis is self-limiting, with skin lesions usually resolving spontaneously within 7–10 days without treatment, whereas the administration of antihistamines or topical corticosteroids can significantly alleviate itching and erythema within 3–5 days (11, 12). Notably, most cases are associated with exposure to rodents, such as laboratory personnel, pet rodent owners, or residents living in rodent-infested environments. The mites can be transmitted to humans through host migration or environmental contaminants (e.g., rodent nest debris) (15, 33). However, because these mites are nocturnal and extremely small, they are frequently misdiagnosed clinically as insect bites, allergic dermatitis, or bacterial infections. Definitive diagnosis requires a combination of rodent exposure history and microscopic examination (such as the detection of mites in skin scrapings or clothing samples) (12, 33).

Overall, the core clinical feature of *O. bacoti* dermatitis is pruritic erythematous papules (Figure 12), and its prevention and control must incorporate environmental management to interrupt the transmission chain (15). Clinical treatment relies primarily on symptomatic relief, such as antihistamines, while public health measures should focus on rodent host management, including rodent eradication and habitat sanitation (12, 33). Currently, there is no clear evidence in the literature indicating that *O. bacoti* can cause pulmonary acariasis. Pulmonary acariasis is typically caused by mites of the families Acaridae and Glycyphagidae, which differs from the obligate hematophagous habit of *O. bacoti*.

**Figure 12:**
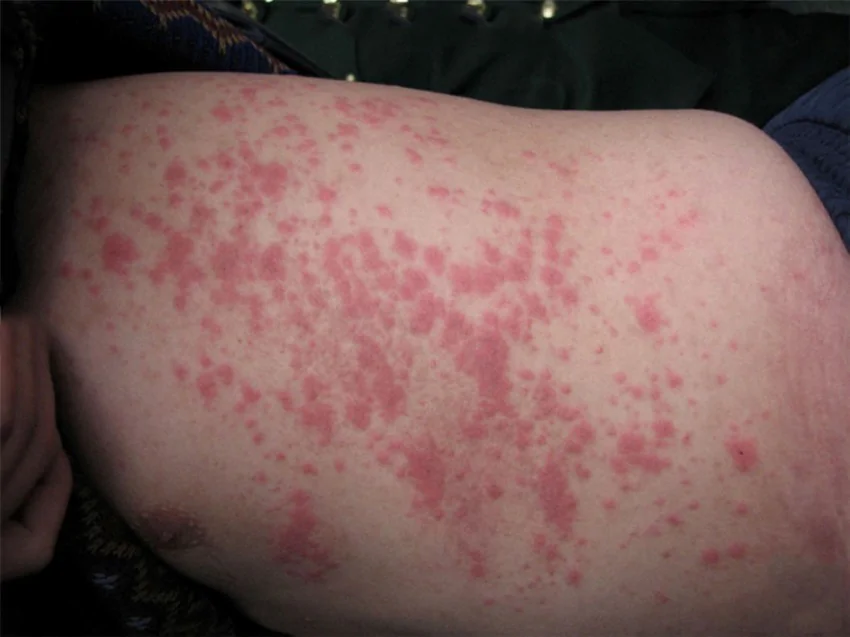
Scattered erythema and papules on a patient’s back caused by the biting of *Ornithonyssus bacoti* [modified from Ma and Hu (83)].

### 6.3 Indirect harms of *Ornithonyssus bacoti*

*Ornithonyssus bacoti* is not only a blood-sucking ectoparasite but also serves as a potential vector or pathogen carrier for various zoonotic pathogens. Evidence of zoonoses related to *Ornithonyssus bacoti* is shown in Table 2. Its indirect harms are primarily concentrated in the following diseases.

**Table 2:** Matrix of zoonotic pathogens associated with *Ornithonyssus bacoti*.

Pathogen agent	Associated disease	Primary host / reservoir	Detection / transmission evidence in *O. bacoti*	Vector competence status	Human transmission proof	Evidence level and grade	Key references
*Rickettsia akari*	Rickettsialpox	*Rattus tanezumi*, *Rattus norvegicus*	Isolation of live bacteria; PCR detection of DNA; salivary gland presence	Confirmed biological vector (transmits via bite)	Confirmed (epidemiological link in urban outbreaks)	Level I (Strong evidence)	Paddock and Eremeeva (38); Reeves et al. (23)
*Litomosoides sigmodontis*	Cotton rat filariasis	*Sigmodon hispidus* (Rodents only)	Larval development within mite coelom; transmission during feeding	Confirmed obligate intermediate host	None (non-pathogenic to humans)	Level I (Strong host evidence, non-zoonotic)	Renz and Wenk (43)
Hantaviruses (Hantaan / Seoul viruses)	Hemorrhagic Fever with Renal Syndrome (HFRS)	*Rattus norvegicus*, *Apodemus agrarius*	Viral RNA detected by RT-PCR; fluorescent antigen in digestive / genital tracts	Experimental rodent transmission via bite demonstrated	Unproven (aerosol and direct contact remain primary)	Level I/II (Moderate evidence for vector potential; human biting transmission unconfirmed)	Song (40); Yu and Tesh (41); Xu et al. (42)
*Bartonella* spp. (e.g., *B. elizabethae*)	Bartonellosis	*Rattus* spp.	DNA amplification by PCR in field-collected mites	Unproven (carries bacterial DNA; competence unconfirmed)	Unproven	Level I (Pathogen carriage confirmed; vector competence unconfirmed)	Reeves et al. (23); de Castro et al. (45)
*Coxiella burnetii* / *Borrelia* spp.	Q fever / Relapsing fever	Rodents, livestock	Molecular / PCR detection of DNA fragments	Unproven (incidental carriage or ingestion with blood meal)	None	Level I/III (Carriage only; no vector capacity proven)	Alghamdi (44)

Level I refers to direct empirical evidence obtained from *Ornithonyssus bacoti*; Level II denotes indirect evidence extrapolated from related gamasid mite species; and Level III represents biological hypotheses that require further empirical validation.

#### 6.3.1 Rickettsialpox

Rickettsialpox is caused by *Rickettsia akari* (an obligate intracellular bacterium of the genus *Rickettsia*), with rodents (such as *Rattus tanezumi* and *Rattus norvegicus*) acting as the primary infectious reservoirs. *O. bacoti* transmits *R. akari* to humans after biting infected hosts. The disease is mainly distributed in urban areas, such as New York (USA), Ukraine, and South Korea, whereas no indigenous cases have been reported in China (29, 35, 36). Laboratory studies have confirmed that after *O. bacoti* feeds on rodents infected with *R. akari*, its saliva contains the pathogen, which can then be transmitted to healthy hosts, thereby demonstrating its vector capacity. Furthermore, in outbreak cases in New York, a large number of *O. bacoti* were found in the living environments of patients, and *R. akari* was detected in host rodents, further supporting its role as a transmission vector (23, 37, 38). Molecular studies have also detected *R. akari* DNA within *O. bacoti*, providing molecular evidence of the association between the pathogen and the mite.

#### 6.3.2 Hemorrhagic fever with renal syndrome

Hemorrhagic fever with renal syndrome (HFRS) is primarily caused by orthohantaviruses, specifically Hantaan virus (HTNV) and Seoul virus (SEOV), with synanthropic and wild rodents (*Rattus norvegicus*, *Apodemus agrarius*) acting as natural reservoirs. While the primary transmission route to humans is the inhalation of virus-containing aerosols from rodent excretions, the potential vector role of mites such as *O. bacoti* warrants investigation. Several studies have provided evidence of viral presence and potential transmission in mites. Hantaviral RNA and fluorescent antigens have been detected in field-collected *O. bacoti* in endemic areas of China, specifically within the mites’ digestive and genital tracts (16, 39–41). Furthermore, laboratory models have demonstrated that *O. bacoti* can transmit the virus to mice via biting (15, 34, 42). However, despite these findings, the natural vector competence and quantitative contribution of *O. bacoti* to human HFRS transmission remain unproven (Level I/II evidence). There is currently no direct evidence confirming that *O. bacoti* causes human infection in natural settings. Therefore, *O. bacoti* should be regarded primarily as an opportunistic mechanical carrier or a secondary amplifier in rodent-to-rodent transmission, rather than a primary vector for humans. The specific transmission mechanisms and ecological impact require further investigation (37, 42).

#### 6.3.3 Cotton rat filariasis

Cotton rat filariasis is caused by the filarial nematode *Litomosoides sigmodontis*, which primarily infects rodents (such as cotton rats), with no human cases reported to date. *O. bacoti* serves as an intermediate host, harboring filarial larvae and transmitting them through biting. Experiments have confirmed that filarial larvae can complete their developmental stages within the mite body and eventually infect new hosts through bites (43). Although this disease poses no direct threat to humans, it reveals the biological characteristics of *O. bacoti* in parasitic transmission and provides a reference for studying other vector-borne diseases.

#### 6.3.4 Other zoonoses

In addition to the previously mentioned diseases, *O. bacoti* may also be associated with *Bartonella* species. Studies have shown that *Bartonella* DNA has been detected in these mites (23), but their transmission capability remains unclear. Moreover, *O. bacoti* can harbor Seoul virus, although its role in transmission is secondary to the aerosol pathway (37, 40, 44). These findings suggest that *O. bacoti* may participate in the ecological maintenance of various pathogens, but its actual public health risks still require further assessment (36, 45–48).

In summary, rickettsialpox represents the primary threat among the indirect harms of *O. bacoti*, with its transmission role well-supported by both laboratory and epidemiological evidence (38). The vector role of *O. bacoti* in transmitting HFRS remains controversial and warrants more research (49). Cotton rat filariasis (*L. sigmodontis* infection) only affects rodents and poses no direct risk to humans, and its role in transmitting other pathogens (such as *Bartonella* spp.) remains to be elucidated (23). Prevention and control strategies should center on suppressing rodent hosts and reducing mite density, while strengthening vector surveillance to block potential transmission pathways.

## 7 Chromosomes and genomic structure of *Ornithonyssus bacoti*

### 7.1 Chromosomes of *Ornithonyssus bacoti*

Previous studies have demonstrated that *O. bacoti* exhibits a haplodiploid sex-determination system common among mites, where females are diploid (2*n* = 16) and males are haploid (*n* = 8) (50). Karyotypic analysis revealed that its autosomes are metacentric or submetacentric, presenting a small morphology without distinct differentiation. The sex chromosomes of *O. bacoti*, however, have not yet been clearly identified, suggesting that the mechanism of sex determination may be associated with a haploid male system (50). This mechanism aligns with the sex-determination pattern observed in most gamasid mites, such as the poultry red mite *Dermanyssus gallinae*, where males develop from unfertilized eggs into haploids, and females develop from fertilized eggs into diploids (17, 50). Nevertheless, the molecular mechanisms underlying sex determination—such as the presence of master sex-determining genes or epigenetic regulation—remain to be further validated through genomic or transcriptomic investigations.

In terms of chromosomal banding patterns, systematic research outcomes are currently lacking. Only a few experiments utilizing Giemsa staining have observed heterochromatic regions at the chromosome termini, without establishing definitive banding patterns. Furthermore, the chromosome number of *O. bacoti* (*n* = 8) differs from that of other species within the genus *Ornithonyssus* (e.g., *Ornithonyssus sylviarum*, *n* = 9), suggesting the potential occurrence of chromosome fusion or fission events within this genus (51, 52).

In summary, the chromosomal characteristics of *O. bacoti* center around a haplodiploid system (*n* = 8, 2*n* = 16), featuring a simple karyotype and undifferentiated sex chromosomes (50). However, conspicuous gaps remain regarding its chromosomal banding, sex-determining genes, and epigenetic regulatory mechanisms. Future studies should leverage advanced molecular biology techniques, such as fluorescence in situ hybridization (FISH) and whole-genome sequencing, to explore these aspects in depth.

### 7.2 Genomic insights and molecular adaptations

To date, genomic studies on *Ornithonyssus bacoti* remain in an introductory phase. Unlike model arthropods, a high-coverage, chromosome-level reference genome assembly for *O. bacoti* has not yet been completed. Current molecular resources are primarily restricted to the complete mitochondrial genome and *de novo* transcriptomic assemblies (53–55). The circular double-stranded mitogenome comprises 37 standard genes (13 protein-coding genes, 22 tRNAs, and 2 rRNAs) alongside an AT-rich control region. Notably, its structural gene order exhibits conserved architectural patterns previously characterized in the related *Ornithonyssus sylviarum*, typical of Mesostigmata (56, 57).

Transcriptomic profiling of *O. bacoti* under post-engorgement states has identified candidate gene families putatively associated with hematophagous digestion, such as cysteine proteases, metalloproteinases, and salivary anticoagulants. These transcripts are hypothesized to assist in blood meal processing and the suppression of host hemostasis (58). In related blood-feeding gamasid mites, notably *Dermanyssus gallinae*, expanded gene clusters of cytochrome P450 monooxygenases, glutathione S-transferases (GSTs), and esterases have been directly linked to metabolic detoxification of synthetic pyrethroids in the related *Dermanyssus gallinae* (59). While transcriptomic orthologs of these detoxification enzymes are present in *O. bacoti*, their explicit functional contribution to clinical acaricide resistance remains a Level II inference that necessitates definitive confirmation through target-site mutation screening and gene knockdown studies in *O. bacoti* specifically (39, 60–62).

## 8 Physiological and biochemical studies of *Ornithonyssus bacoti*

Physiological and biochemical investigations into *O. bacoti* have shed light on its genetic diversity and metabolic adaptation mechanisms. Electrophoretic analyses of esterase isoenzymes (EST) and malate dehydrogenase (MDH) revealed significant variations in enzyme profiles among different geographical populations. These patterns parallel those observed in comparative population studies of *Ornithonyssus sylviarum*, suggesting that genetic diversity in this lineage is shaped by host adaptation or environmental selection pressures (63, 64). For example, populations from different regions in China displayed polymorphisms in EST isoenzyme profiles, implying that geographical isolation or host divergence may drive genetic differentiation. Moreover, the isoenzyme profiles demonstrated that the metabolic enzyme activities of *O. bacoti* fluctuate significantly across different developmental stages (nymphs versus adults) and nutritional states (pre- versus post-blood-feeding). Following a blood meal, the activity of enzymes associated with energy metabolism (such as MDH) increased markedly, indicating that its metabolic network can be dynamically reconfigured to accommodate blood-feeding behaviors (62, 65).

These discoveries not only clarify the population genetic structure of *O. bacoti* but also elucidate its strategy for adapting to a hematophagous lifestyle through metabolic plasticity. Future endeavors should integrate metabolomics with gene-editing technologies (such as CRISPR) to further resolve its physiological regulatory networks and environmental adaptation mechanisms, particularly the dynamic relationship between gene expression and enzyme activity.

## 9 Artificial cultivation of *Ornithonyssus bacoti*

Research on the artificial cultivation methods of *O. bacoti* mainly focuses on optimizing rearing conditions to enhance reproductive efficiency, maintain population stability, and meet experimental demands. Among conventional rearing methods, in vitro rearing using plaster bottles combined with animal blood membranes (such as cattle, sheep, or rodent blood) can provide an appropriate humid environment to support mite survival and oviposition in the short term. However, long-term rearing suffers from a decline in survival rate and a prolonged reproductive cycle due to the lack of host physiological signals (e.g., body temperature and carbon dioxide) (66). In contrast, live hosts (e.g., mice) provide stable temperature, humidity, and blood sources, significantly enhancing the reproductive efficiency of the mites. Specifically, the egg production of female mites increases after blood-feeding, and the developmental period from nymphs to adults is shortened, although host immune responses can moderate mite density over prolonged infestations in *O. sylviarum* systems (67, 68).

In the exploration of specialized rearing methods, the application of alternative hosts (such as hamsters and guinea pigs) indicates that *O. bacoti* has a broad host adaptability, though reproductive efficiency varies significantly across host species (34, 36). Synthetic diets (containing hemoglobin, amino acids, and vitamins) can partially replace natural blood, but host-specific chemical signals (e.g., lactic acid and adenosine triphosphate) must be added to stimulate blood-feeding behavior (69, 70). However, the long-term rearing efficacy of artificial diets remains inferior to that of live hosts. Furthermore, the optimization of environmental parameters is crucial. The control of temperature and humidity (25–30 °C, 70–80% RH) along with dark or dim light conditions can significantly improve mite development and oviposition efficiency, whereas high temperatures (>35 °C) or low humidity (<50%) lead to a sharp decline in survival rates (71, 72).

Overall, the artificial cultivation of *O. bacoti* requires a balance between environmental control and the simulation of host signals. Although live hosts (e.g., white mouse) offer optimal conditions, they are associated with high costs; in vitro methods (e.g., plaster bottles combined with blood membranes) are straightforward to operate but require improvements in diet composition to enhance long-term stability (73). Future research could integrate gene-edited hosts or synthetic biology technologies to develop more efficient and ethically compliant rearing systems.

## 10 Integrated One Health framework for *Ornithonyssus bacoti* surveillance and management

The persistence of *O. bacoti* at the interface of human habitations, laboratory vivaria, and wild animal reservoirs underscores the imperative for a One Health approach. Effective long-term suppression cannot rely solely on isolated chemical spraying or symptomatic medical treatment, but demands multi-sectoral collaboration integrating human health, veterinary medicine, wildlife ecology, and environmental engineering. The efficacy, application scenarios, and resistance evidence of *Ornithonyssus bacoti* control drugs are shown in Table 3.

**Table 3:** Evaluation of chemical and integrated control measures against *Ornithonyssus bacoti.*

Chemical / control class	Active ingredient / method	Target life stage	Application context	Efficacy and outcome	Resistance evidence and limitations	Evidence level	Key references
Synthetic Pyrethroids	Permethrin, Lambda-cyhalothrin	Protonymphs, Deutonymphs, Adults	Environmental residual spray; bedding treatment in animal vivaria	High acute mortality (>95% reduction within 14 days)	Suspected field resistance in prolonged vivarium usage among adults/nymphs; eggs display physical impermeability to non-systemic contact sprays	Level I	Hill et al. (77); Clancy et al. (69)
Organophosphates	Malathion	Nymphs, Adults	Structural spray in host harborage areas	Moderate to high contact toxicity	High mammalian toxicity; potential cross-resistance; restricted indoor use	Level I/II	Hill et al. (77)
Isoxazolines	Fluralaner	Feeding Nymphs, Adults	Systemic administration to rodent hosts (experimental/pet)	High mortality (>98%) following blood meal	High efficacy; requires host feeding; extrapolations partly from *O. sylviarum* models	Level II	Mullens et al. (78)
Rodenticides (Host Reduction)	Bromadiolone, Brodifacoum	Rodent hosts (*Rattus* spp.)	Community and vivarium rodent eradication campaigns	Indirect population collapse of mites due to host elimination	Mites migrate away from dead hosts; must combine with simultaneous acaricide spraying	Level I	Yin et al. (80)
Environmental Control	Sanitation, crevice sealing, bedding disposal	All stages (including eggs)	Vivarium management, urban housing sanitation	Disrupts breeding harborage and egg oviposition	Non-chemical; requires continuous compliance	Level I	Watson (22); Sargison et al. (76)

The operational interactions across sectors within this integrated framework are detailed in Table 4. First, environmental management serves as the foundation, which disrupts the breeding environment of mites by eliminating the habitats (e.g., refuse accumulation areas) of hosts such as rodents (22, 36). Second, host control is critical; reducing host density through rodent eradication (using toxic baits and rodent traps) can indirectly diminish mite populations (74–76). Integrated control of *O. bacoti* relies on complementary chemical and physical strategies. Chemical control primarily involves the targeted application of conventional acaricides, such as pyrethroids (e.g., permethrin) and organophosphates (e.g., malathion), to host nests and human activity areas (69, 77, 78). Novel systemic agents, particularly isoxazolines, have demonstrated high contact toxicity against avian mesostigmatid mites (*O. sylviarum*) (78); however, their direct applicability to *O. bacoti* remains a Level II extrapolation requiring empirical validation in rodent facilities. In parallel, physical protective measures, including protective clothing, DEET-containing repellents, and sealing architectural crevices, can effectively minimize human–mite contact (79).

**Table 4:** One Health conceptual framework for *Ornithonyssus bacoti* management.

One Health domain	Core intervention sector	Operational mechanism against *O. bacoti*	Cross-sectoral interface
Human health	Clinical dermatitis diagnosis and treatment and personal protection	Early differential diagnosis; topical corticosteroid application for pruritus relief; use of DEET (N, N-diethyl-meta-toluamide) mosquito repellent for personal protection	Notification mechanism between dermatology departments and public health disease control agencies
Animal health	Vivarium management: Experimental animals and pets	Quarantine and isolation; targeted deworming using pyrethroids or isoxazoline-class compounds	Joint monitoring between veterinary departments and experimental animal centers
Vector ecology	Rodent mite density monitoring and acaricide resistance monitoring	Host nest exploration; acaricide sensitivity baseline determination; egg-stage barrier targeted medication	Collaboration between entomology research institutions and vector biology laboratories
Environmental health	Host habitat disruption and environmental sanitation	Garbage clearance; sealing of building crevices and fissures; synchronized rodent control and environmental residual insecticide spraying	Coordination between municipal sanitation services and community pest control operations (PCO) teams

Successful cases demonstrate that integrated measures can significantly enhance control efficiency. For instance, following a laboratory infestation outbreak, a combination of rodent eradication (bromadiolone toxic baits) and permethrin spraying resulted in a 98% reduction in mite density within two weeks (22, 69, 77). An urban community in China implemented refuse cleanup, rodent control campaigns (brodifacoum toxic baits), and indoor residual spraying (lambda-cyhalothrin); within three months, the rodent infestation rate decreased by 85%, and mite bite cases were completely eliminated (80). In another case, a zoo successfully disrupted the transmission chain through environmental cleaning (removing bedding material), host isolation (enclosed management of rodent exhibition areas), and localized thermal fogging treatment (imidacloprid) (69, 77). These practices validate the synergistic effects of environmental management and host regulation (67, 69, 77).

## 11 Conclusion

In conclusion, the prevention and control of *O. bacoti* should be founded on environmental management, centered on host control, and supplemented by chemical control, with integrated strategies yielding the best results. Future attention must be directed toward insecticide resistance monitoring and the development of eco-friendly technologies (such as biopesticides) to address long-term control challenges.
