# Supplementary material for: Ornithonyssus bacoti (tropical rat mite): a critical review of biology, vector potential, and integrated management
Source: Front Vet Sci. 2026 Oct 13;13:1954092. doi: 10.3389/fvets.2026.1954092 (PMC13711307; doi:10.3389/fvets.2026.1954092)
Supplement: Supplementary file 1 [file Table_1.docx]

Supplementary Table 1. Global georeferenced occurrence and biogeographic distribution points of *Ornithonyssus bacoti*.

| Point_ID | Country | Locality / Province | Temporal Status | Data_Source |
| --- | --- | --- | --- | --- |
| OB-001 | Australia | Gulargambone / Galargambone | Historical | GBIF occurrence record |
| OB-002 | Australia | Maria Island (National Park) | Historical | GBIF occurrence record |
| OB-003 | Australia | intercepted at Mile End | Historical | GBIF occurrence record |
| OB-004 | Australia | Western Australia | Historical | GBIF occurrence record |
| OB-005 | Australia | South Perth, Perth Zoo | Recent | GBIF occurrence record |
| OB-006 | Brazil | Barro Alto, R. San Francisco | Historical | GBIF occurrence record |
| OB-007 | Brazil | Minas Geraes, Barro Alto, Rio San Francisco | Historical | GBIF occurrence record |
| OB-008 | Canada | Montreal, Quebec | Historical | GBIF occurrence record |
| OB-009 | Canada | British Columbia | Recent | GBIF occurrence record |
| OB-010 | Canada | Ontario | Recent | GBIF occurrence record |
| OB-011 | Chile | Barrio Universitario, Universidad de Concepcion | Recent | GBIF occurrence record |
| OB-012 | Chile | Guafo Island, Northern Chilean Patagonia | Recent | Seguel et al. (2017) |
| OB-013 | China | Yunnan (Dali) | Recent | Yin et al. (2021) |
| OB-014 | Democratic Republic of the Congo | Kikanda | Historical | GBIF occurrence record |
| OB-015 | Democratic Republic of the Congo | Kiswishi | Historical | GBIF occurrence record |
| OB-016 | Democratic Republic of the Congo | Lwiro | Historical | GBIF occurrence record |
| OB-017 | France | Unspecified locality | Recent | GBIF occurrence record |
| OB-018 | Germany | Kamenz (Kamenz) | Historical | GBIF occurrence record |
| OB-019 | Germany | Lubeck | Historical | Engel et al. (1998) |
| OB-020 | India | Nilgiris, Tamil Nadu | Recent | Bhuyan & Nath (2016) |
| OB-021 | Iraq | Baghdad, Camp Victory | Recent | GBIF occurrence record |
| OB-022 | Italy | Southern Italy (Campania) | Recent | d'Ovidio et al. (2018) |
| OB-023 | Italy | Veneto | Recent | GBIF occurrence record |
| OB-024 | Mexico | Col. Chepevera | Historical | GBIF occurrence record |
| OB-025 | Niue | Niue Island, Maila | Historical | GBIF occurrence record |
| OB-026 | Papua New Guinea | Kila Kila (Number1 National Capital) | Historical | GBIF occurrence record |
| OB-027 | Peru | Bolognesi, Huayllacan | Historical | GBIF occurrence record |
| OB-028 | Sweden | Eslov | Historical | GBIF occurrence record |
| OB-029 | Switzerland | Zurich | Recent | Fiechter et al. (2011) |
| OB-030 | Ukraine | Chernivetska Oblast | Historical | GBIF occurrence record |
| OB-031 | United Kingdom | Edinburgh, Scotland | Recent | Sargison et al. (2025) |
| OB-032 | United States | New York City | Historical | GBIF occurrence record |
| OB-033 | United States | Palmer, 8 km NNE | Historical | GBIF occurrence record |
| OB-034 | United States | Decatur Co., 6 mi. E. Lamoni | Historical | GBIF occurrence record |
| OB-035 | United States | Phoenix, Maricopa Co., AZ | Historical | GBIF occurrence record |
| OB-036 | United States | Washington, National Zoological Park | Recent | GBIF occurrence record |
| OB-037 | United States | Lab colony at Harvard University, Cambridge | Recent | GBIF occurrence record |
| OB-038 | United States | California | Recent | GBIF occurrence record |
| OB-039 | China | Shanghai | Unresolved | Compilation of Chinese literature records |
| OB-040 | China | Yunnan: Kunming | Historical | He 1999; Luo et al. 2007 |
| OB-041 | China | Beijing | Unresolved | Compilation of Chinese literature records |
| OB-042 | China | Sichuan: Chengdu | Historical | Luo et al. 1985; Yang et al. |
| OB-043 | China | Ningxia: Yinchuan | Historical | Cai et al. 1994 |
| OB-044 | China | Anhui: Hefei | Unresolved | Anhui Province Gamasid Mite Survey |
| OB-045 | China | Shandong: Jinan | Recent | Zhao et al. 2008, 2012 |
| OB-046 | China | Shanxi: Taiyuan | Historical | Tian 1984 |
| OB-047 | China | Guangdong: Guangzhou | Unresolved | Compilation of Chinese literature records |
| OB-048 | China | Guangxi: Nanning | Unresolved | Liang et al. |
| OB-049 | China | Jiangsu: Nanjing | Unresolved | Compilation of Chinese literature records |
| OB-050 | China | Jiangxi: Nanchang | Unresolved | Compilation of Chinese literature records |
| OB-051 | China | Henan: Zhengzhou | Unresolved | Compilation of Chinese literature records |
| OB-052 | China | Zhejiang: Hangzhou | Unresolved | Zhejiang Province Gamasid Mite Checklist 2017 |
| OB-053 | China | Hainan: Haikou | Unresolved | Compilation of Chinese literature records |
| OB-054 | China | Hubei: Wuhan | Unresolved | Compilation of Chinese literature records |
| OB-055 | China | Hunan: Changsha | Unresolved | Compilation of Chinese literature records |
| OB-056 | China | Fujian: Fuzhou | Unresolved | Strait Journal of Preventive Medicine 2017 (Xiamen survey) |
| OB-057 | China | Guizhou: Guiyang | Historical | He 1988; Mo et al. 1994 (Qiandongnan 7 counties) |
| OB-058 | China | Liaoning: Shenyang | Historical | Sun et al. 1993 |
| OB-059 | China | Chongqing | Unresolved | Compilation of Chinese literature records |
| OB-060 | China | Heilongjiang: Mudanjiang | Recent | Zhang et al. 2000 |
| OB-061 | China | Inner Mongolia: Hohhot | Historical | Sun 1983 (Chinese Journal of Parasitology and Parasitic Diseases) |
| OB-062 | China | Shaanxi: Xi'an | Recent | Chinese Journal of Vector Biology and Control 2017 (First report in Xi'an) |
| OB-063 | Finland | Pirkanmaa | Unresolved | GBIF occurrence record |
| OB-064 | Iran | Tehran | Recent | Telmadarraiy et al. (2007); Mohebali et al. (1997); Hanafi-Bojd et al. (2007) |
